# Proflavine Hemisulfate as a Fluorescent Contrast Agent for Point-of-Care Cytology

**DOI:** 10.1371/journal.pone.0125598

**Published:** 2015-05-11

**Authors:** Sandra P. Prieto, Amy J. Powless, Jackson W. Boice, Shree G. Sharma, Timothy J. Muldoon

**Affiliations:** 1 Biomedical Engineering Department, University of Arkansas, Fayetteville, Arkansas 72701, United States of America; 2 10810 Executive Center Dr., Nephropath Ste. 100, Little Rock, Arkansas 72211, United States of America; Queen Mary Hospital, HONG KONG

## Abstract

Proflavine hemisulfate, an acridine-derived fluorescent dye, can be used as a rapid stain for cytologic examination of biological specimens. Proflavine fluorescently stains cell nuclei and cytoplasmic structures, owing to its small amphipathic structure and ability to intercalate DNA. In this manuscript, we demonstrated the use of proflavine as a rapid cytologic dye on a number of specimens, including normal exfoliated oral squamous cells, cultured human oral squamous carcinoma cells, and leukocytes derived from whole blood specimens using a custom-built, portable, LED-illuminated fluorescence microscope. No incubation time was needed after suspending cells in 0.01% (w/v) proflavine diluted in saline. Images of proflavine stained oral cells had clearly visible nuclei as well as granular cytoplasm, while stained leukocytes exhibited bright nuclei, and highlighted the multilobar nature of nuclei in neutrophils. We also demonstrated the utility of quantitative analysis of digital images of proflavine stained cells, which can be used to detect significant morphological differences between different cell types. Proflavine stained oral cells have well-defined nuclei and cell membranes which allowed for quantitative analysis of nuclear to cytoplasmic ratios, as well as image texture analysis to extract quantitative image features.

## Introduction

Cytology is defined as the study of cells on a microscopic scale with a focus on morphology and structural distinctions [1]. Considering the many cytological procedures that depend on visual diagnosis, and distinguishing of structural features such as nuclear size, nuclear to cytoplasmic ratios, and cytoplasmic features, the field depends significantly on contrast-enhancing agents. Clinical cytology is typically performed by sample collection, fixation, staining, and visual inspection by a trained pathologist. Cytology specimens may include peripheral blood smears, fine needle aspirations (FNA), bronchial alveolar lavages (BAL), and oral as well as vaginal/cervical exfoliated cells. Clinical cytology provides complementary information to more conventional histopathology examination of thin tissue sections made from solid tissue, particularly when screening for cellular atypia and dysplasia [2].

Common contrast agents used for cytology include conventional hematoxylin and eosin (H&E), Giemsa, and Papanicolaou (PAP) staining [3,4]. Fluorescent dyes are also popular contrast agents due to their high signal-to-background ratios, as well as offering the option for varying levels of specificity in targeting of molecules and/or structures. Fluorescent contrast agents do not necessarily need to be as target-specific as immunofluorescent staining. Common untargeted exogenous fluorescent stains include ethidium bromide, acridine orange, propidium iodide, and acriflavine hydrochloride / proflavine hemisulfate. Popular stains such as the fluorescent dye acridine orange are widely used in flow cytometry and cytochemistry applications which do not require the selectivity of immunofluorescence, such as the lysosome staining of living blood cells, and in distinguishing between different types of lung cells [5]. Nucleic acid probes, which do not fall under the category of target-specific dyes, despite their preferential binding to nucleic acids, are used routinely to determine the ploidy or phase of a cell, but usually require fixation and permeabilization [6,7,8]. A common benefit of untargeted fluorescent stains is the simplicity of the staining process, compared to the hybridization time and environmental factors, such as temperature and media, required for immunolabeling.

Proflavine, an aminoacridine-derived dye similar to acridine orange, non-specifically stains cellular structures, as well as preferentially stains cell nuclei. It exhibits fluorescence with a peak excitation around 460nm and emission around 515nm, with a quantum efficiency of 0.5 [9,10]. Proflavine can be used to rapidly stain fresh cells due to its small molecular size and amphipathic chemistry, enabling the molecule to easily pass through the lipid bilayer of the cell and nuclear membrane [11]. It has been shown to intercalate double stranded DNA, providing strong nuclear contrast [12,13]. In addition, proflavine exhibits less prominent staining of cytoplasmic structures, which can be beneficial for cell classification, allowing for applications in cytological analysis.

Proflavine has been used clinically since 1917 [14], but its use as a cytological tool isn’t widespread despite its numerous advantages. Its ability to intercalate DNA has provided many applications including anti-cancer, anti-bacterial, and anti-viral drugs [15,16,17]. Common research applications include endoscopy and microendoscopy imaging, modalities that benefit from contrast agents that allow for rapid topical staining of intact epithelium, where proflavine is used as a topical contrast agent for histological analysis [14,18,19]. There has also recently been renewed interest in proflavine for point-of-care diagnostic applications in low-resource settings due to its physical and chemical stability in solution, lasting at least 12 months under refrigeration [20].

In this paper we will demonstrate the benefits of proflavine staining in cytological samples, including rapid and preferential staining of nucleic structures such as cytosol, membrane, granules and nuclei. Proflavine’s rapid staining eliminates time-consuming procedures while retaining the benefits of fluorescence imaging. We have demonstrated proflavine staining in a variety of cell types, including normal exfoliated oral squamous cells, an *in vitro* cultured oral squamous carcinoma cell line (Cal 27), and normal human leukocytes from whole blood. All proflavine fluorescence images were acquired using a custom-built, cost-effective and portable fluorescence microscopy platform. Automated image analysis tools were used to demonstrate the feasibility of using computer aided diagnostic approaches on proflavine-stained cytology specimens.

## Materials and Methods

### Mammalian Cell Culture

CAL 27, a squamous cell carcinoma cell line (ATCC CRL-2095, ATCC, Manassas, VA), was cultured in Dulbecco’s Modified Eagle Medium (DMEM) media supplemented with 10% fetal bovine serum (ATCC, Manassas, VA) and 1% Penicillin-Streptomycin (Life Technologies, Grand Island, NY) and incubated at 37°C in 5% CO_2_. The cells were utilized up to passage number four. Before staining, the cells were trypsinized with 0.25% Trypsin-EDTA (Life Technologies, Grand Island, NY).

### Human Cell Collection

All human cells used in this study were collected following written informed consent from healthy volunteers as part of a protocol (IRB#13-06-759) approved by the University of Arkansas Institutional Review Board.

Exfoliated oral epithelial cells were collected through an oral rinse consisting of a 30 sec rinse to remove food particles and bacteria that were discarded followed by a 90 sec collected rinse. The cell suspension was filtered through a 100μm cell strainer (Falcon, Bedford, MA) to remove mucus and washed three times by centrifuging at 200g for 5 min, decanting the supernatant, and resuspending in phosphate buffered saline (1x PBS, Sigma-Aldrich, St. Louis, MO). Whole blood was collected using a fingerstick lancet (30 Gauge Ultra-Thin Lancets, ReliOn, Bentonville, AR). The first drop was discarded [21], and approximately 20μl of blood was collected using a micropipette.

### Papanicolaou Staining of Oral Epithelial Cells

A cotton swab was used to transfer exfoliated oral epithelial cells from the oral cavity to a clean slide, where it was left to dry completely. Following a conventional Papanicolaou staining procedure [22], the cells were fixed with 95% ethanol, chromatin stained using Gill Hematoxylin #1 (Millipore, Billerica, MA), blued with Scott’s tap water substitute (Millipore, Billerica, MA), cytoplasmic counterstained using Modified OG-6 and Modified EA (Millipore, Billerica, MA), and cleaned with Xylene. After mounting a no.1 coverslip, the slides were imaged in brightfield using a Nikon Eclipse Ci microscope (Nikon Instruments Inc., Melville, NY) and a 60x Nikon Plan Apo VC oil immersion objective (N.A. = 1.40, Nikon Instruments Inc., Melville, NY).

### Papanicolaou Staining of CAL 27 Cells

After passaging the CAL 27 cells, the cell suspension, ranging from 600,000–800,000 cells, was centrifuged at 200g for 5 min and the media removed. The cells were resuspended in 1X PBS and washed once by centrifuging at 200g for 5 min, decanting the supernatant, and resuspending in PBS. A glass slide was prepared by pipetting 10 μl of the cell suspension and using the pipette tip to spread the droplet into a monolayer. The slide was dried completely and stained following the same modified Papanicolaou staining procedure described previously. The slide was imaged in brightfield using the same Nikon microscope and 60x oil immersion objective.

### Giemsa Staining of Blood Smears

A peripheral blood smear was Giemsa stained following standard methods[21]. The smear was allowed to completely dry before being fixed with methanol for a few seconds. The slide was then stained with a 10% Giemsa (Alfa Aesar, Ward Hill, MA) solution for 5–10 min. The slide was rinsed with clean water and allowed to dry before mounting a coverslip. The slides were imaged with a brightfield microscope (Nikon Eclipse Ci, Nikon Instruments Inc., Melville, NY) using a 60x oil immersion objective (60x Nikon Plan Apo VC, Nikon Instruments Inc., Melville, NY).

### Proflavine Staining of Oral Epithelial Cells

The samples of exfoliated cells were washed in PBS three times and incubated in 1% albumin from bovine serum (BSA, Sigma-Aldrich, St. Louis, MO) for 5 min. The BSA was removed and the cells were stained with a solution of 0.01% (w/v) proflavine in PBS. Additionally, to collect immature oral epithelial cells, a cotton swab was used to collect normal oral cells from a volunteer. The swab was mixed in a 0.01% (w/v) proflavine solution to suspend and stain the cells. A 10μl sample of stained cells from each collection method was added to a slide and (no.1) coverslipped. The slide was imaged with our epi-illuminated fluorescence system using a 40x Nikon Plan Achromat air objective (N.A. = 0.65, Nikon Instruments Inc., Melville, NY).

### Proflavine Staining of CAL 27

Passaged CAL 27 cells (up to passage number four) were centrifuged at 200g for 5 min and the media removed. The cells were washed once with PBS by centrifuging at 200g for 5 min, decanting the supernatant, and resuspending in 1% BSA for 5 min. The BSA was removed and the cells were stained with the 0.01% (w/v) proflavine solution. The slide preparation and imaging was conducted the same as previously described.

### Proflavine Staining of Leukocytes

The samples of whole blood were stained at a final concentration of 0.01% (w/v) proflavine solution. The slide preparation and imaging was conducted the same as previously described.

### Custom Fluorescence Imaging System

**Fig 1:**
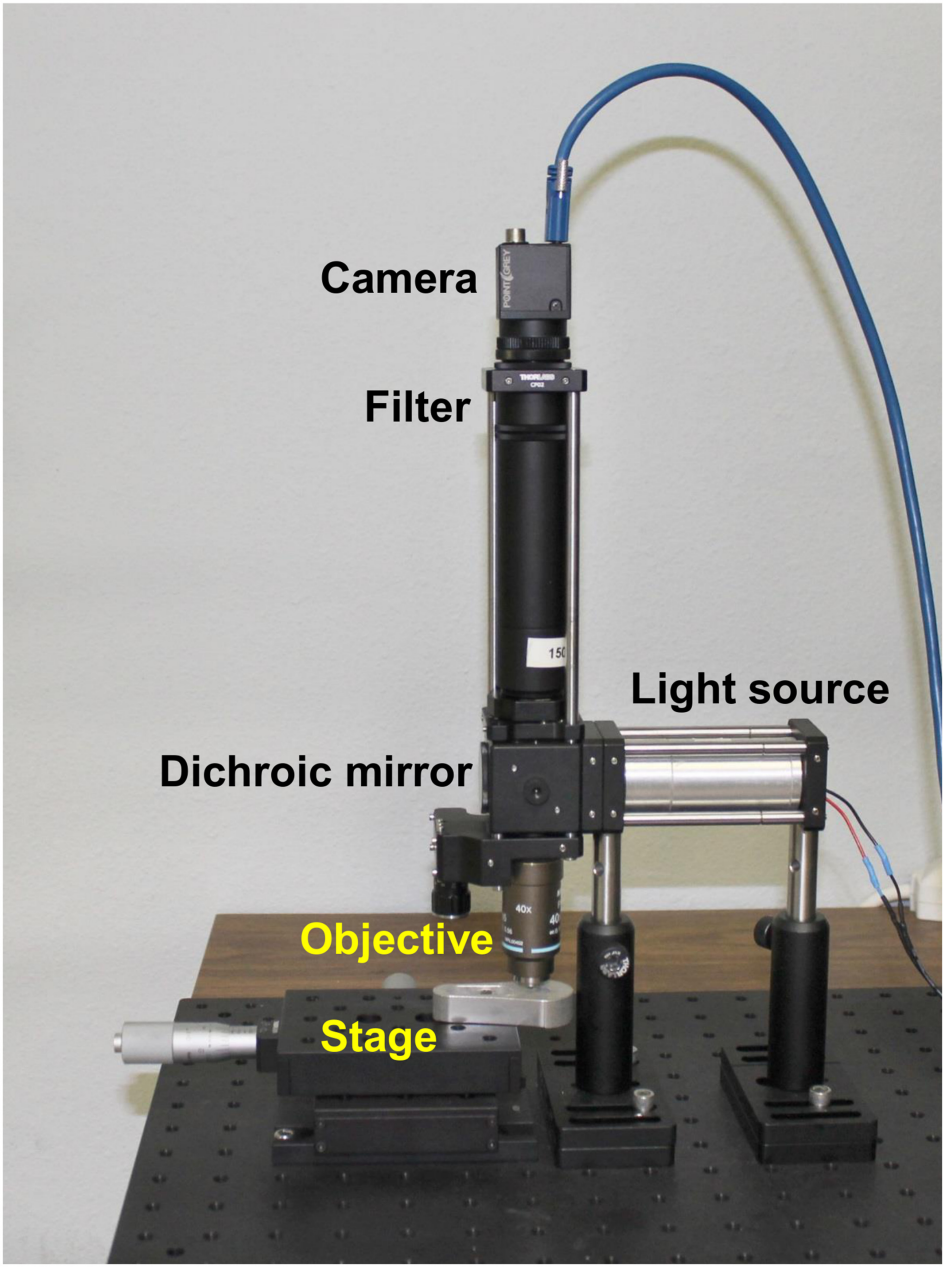
Custom epi-fluorescence microscopy system. Comprises a Flea 3 USB camera, 525/40nm bandpass emission filter, 475nm dichroic filter set, 455nm LED light source, 40x Nikon Plan Achromat air objective and two linear translation stages.

Image acquisition was also performed using a custom epi-fluorescence microscope (see Fig 1) based on a microendoscopy platform previously demonstrated [23]. This system comprises an LED illumination source at a center wavelength of 455nm (Philips, San Jose, California) providing approximately 8mW at the sample, a 475nm dichroic filter set (Chroma Technologies, Bellows Falls, VT) and a 525/40nm bandpass emission filter. The imaging system used a 40x Nikon Plan Achromat air objective (N.A. = 0.65, Nikon Instruments Inc., Melville, NY) and a 150mm achromatic doublet tube lens (Thorlabs, Newton, NJ). A Flea 3 USB camera (Point Grey Research, BC, CA) was used for imaging. The samples were placed on a set of linear translation stages (Thorlabs, Newton, NJ) for x and y axis translation. This system performs comparably to any conventional epi-fluorescence microscope equipped with a fluorescein or green fluorescent protein (GFP) filter set and monochrome camera. Images were taken with no gain, and with exposure times in the range of 100 to 150 msec. Fluorescence images were normalized to ensure equal pixel intensity maxima in all images, in order to compensate for photobleaching and other uneven illumination artefacts. The histograms were stretched, assigning the highest pixel value to be at maximum intensity (255 for an 8-bit image) and the other pixel values were scaled accordingly to conserve the ratios.

### Manual Segmentation for Nuclear to Cytoplasmic Ratio

**Fig 2:**
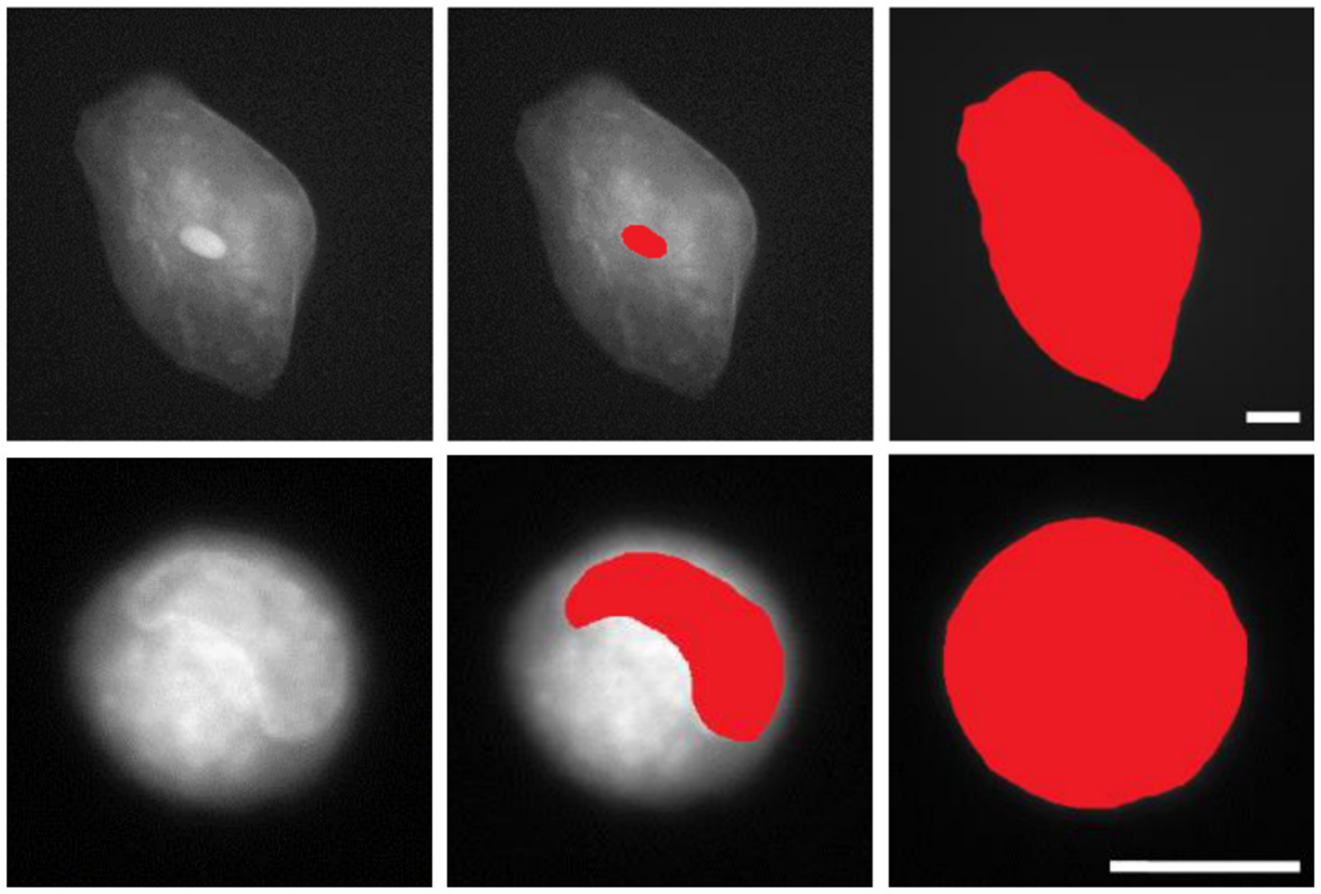
Illustration of manually-segmented oral epithelial cells stained with proflavine. Top row: Normal exfoliated oral epithelial cells. Bottom row: CAL 27 oral squamous carcinoma cells. Scale bars = 10μm.

Images were semi-quantitatively analyzed using ImageJ [24]. Digital images acquired using our custom microscopy system were hand-segmented using ImageJ to isolate the nucleus from the cytoplasm. The boundary around the nucleus was traced using the free-hand tool, measuring the area, and repeating the process for the boundary around the entire cell (see Fig 2). The number of pixels within each measured area (nuclear, whole cell) was tabulated. The cytoplasmic area was then calculated by subtracting the nuclear area from the whole cell. The nuclear to cytoplasmic ratios were then calculated for each cell. The mean and standard deviation values of these ratios were computed for both normal oral and Cal 27 cells.

Image texture features used to analyze the images included the calculated standard deviation of the pixel intensity across the entire image, as well as entropy values. Entropy is an image texture function in the MATLAB toolbox which examines the entire image for pixel intensity variation, as a measurement of heterogeneity in an image. Standard deviation and entropy of one hundred images of each type, normal exfoliated oral squamous epithelial cells and cultured Cal 27 oral squamous carcinoma cells, were computed, averaged, and tabulated. The statistical significance for each group (p values) were computed using a two tailed Student’s t test.

## Results

### Comparison of Cytologic Features Seen in Papanicolaou and Proflavine Stained Oral Cells

**Fig 3:**
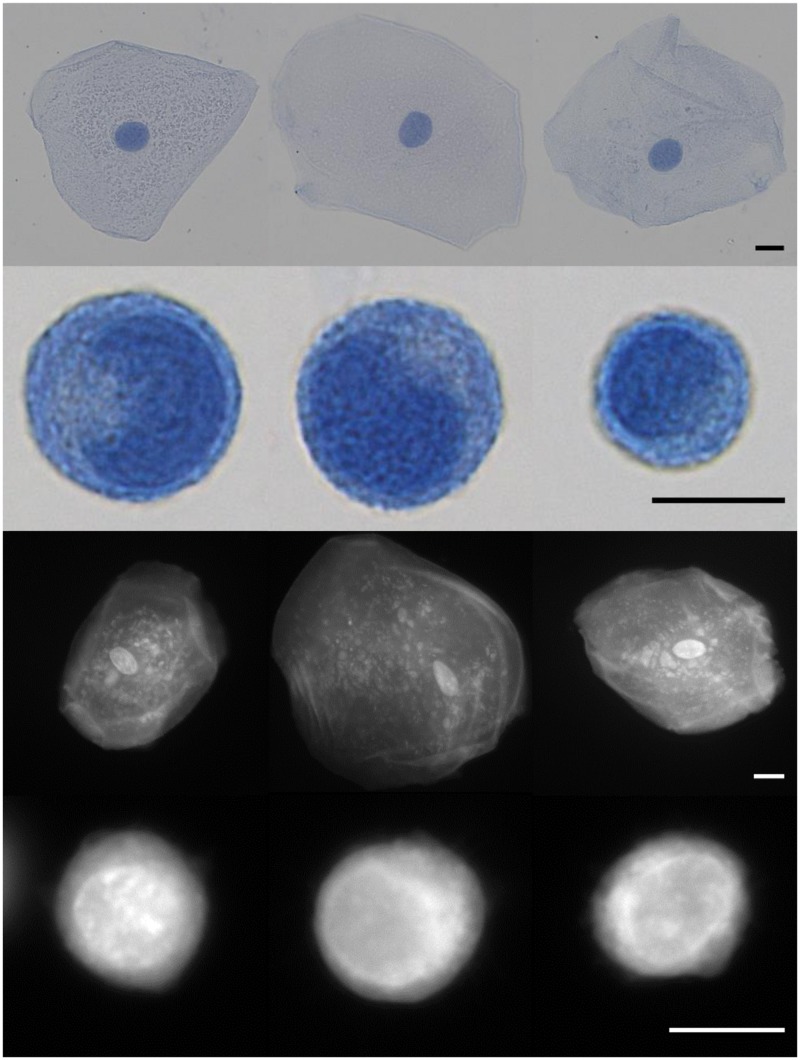
Visual diagnosis comparison of Papanicolaou and proflavine-stained normal oral cells and CAL 27 cell line. First row: Papanicolaou stained normal oral cells. Second row: Papanicolaou stained CAL 27 cells. Third row: Fluorescent images of proflavine stained normal oral cells. Fourth row: Fluorescent images of proflavine stained CAL 27 cells. Scale bars = 10 μm.

**Fig 4:**
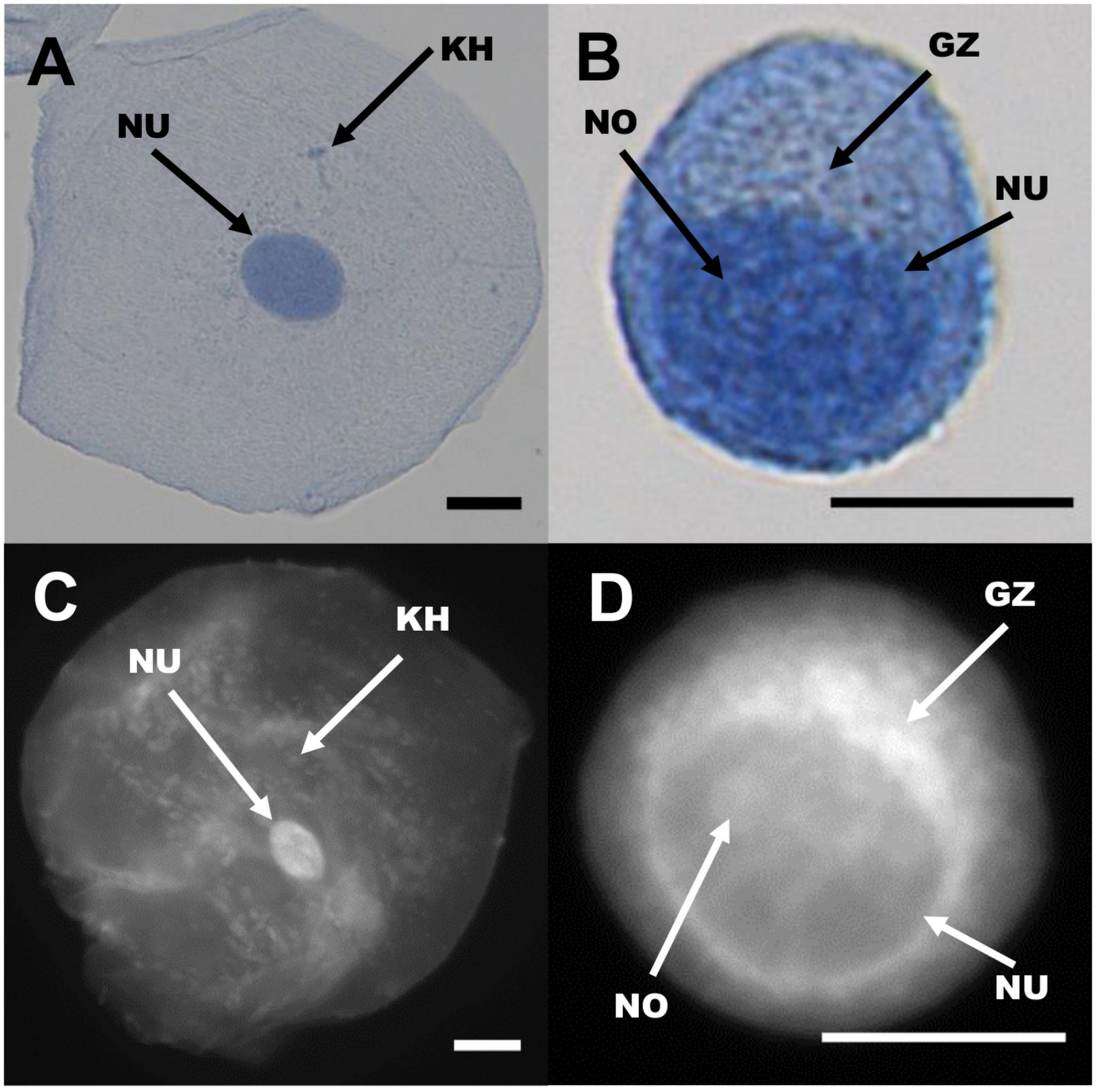
Comparison of cellular features seen in Papanicolaou (top row) and proflavine (bottom row) stained cells. Left column, oral squamous epithelial cells. Right column, Cal 27 squamous carcinoma cells. NU: Nucleus, NO: Nucleolus, KH: Keratohyalin granule, GZ: Golgi zone. Scale bars = 10 μm.

Proflavine stains both cytosol and nuclei in oral cells, yet the two regions are still distinguishable. As seen in Fig 3, Papanicolaou stained cells are compared with similar cell types stained with proflavine. Nuclear as well as cytoplasmic shape, size, granulation (keratohyalin) and the golgi zone are distinguishable in both the colorimetric and fluorescently stained cell types (Fig 4). Note the small, well-defined nuclei in normal oral cells, which exhibit significantly brighter fluorescence than the surrounding cytoplasm. Cytoplasmic detail, such as keratohyalin granules, is also apparent. Cal 27 oral squamous carcinoma cells, by comparison exhibit very large and somewhat undefined nuclear envelopes and prominent Golgi zones. In both cases, proflavine fluorescence and Papanicolaou staining patterns are similar.

### Comparison of Cytologic Features Seen in Giemsa and Proflavine Stained Human Leukocytes

**Fig 5:**
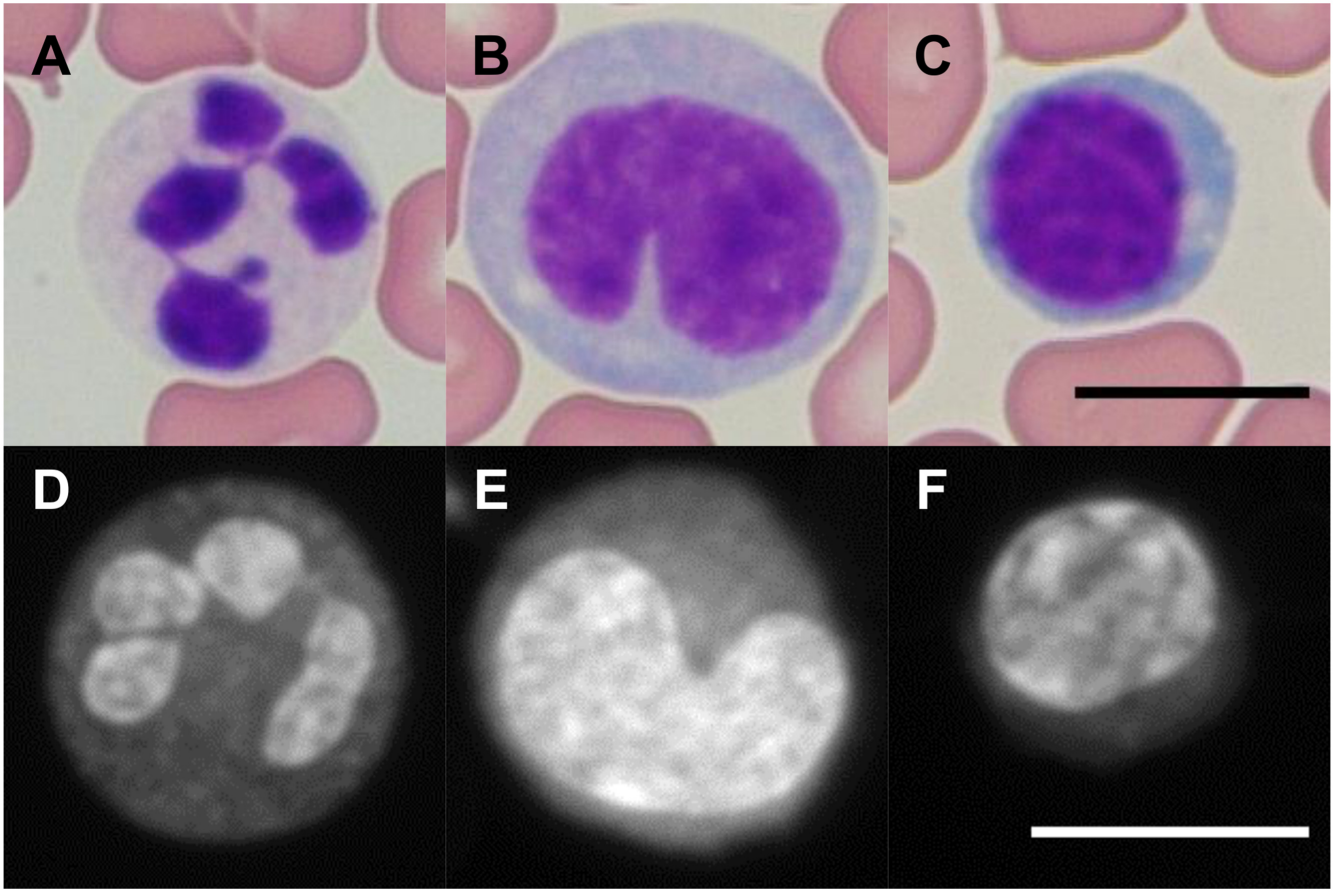
Giemsa (top row) and proflavine (bottom row) stained leukocytes. First row, Giemsa stained neutrophil (A), monocyte (B), lymphocyte (C). Second row, proflavine stained neutrophil (D), monocyte (E), lymphocyte (F). Images were captured at 60X. Fluorescence images were normalized as described in the Methods section. Scale bars = 10μm.

As seen in Fig 5, while Giemsa stains both erythrocytes and leukocytes, proflavine preferentially stains the nuclei-containing leukocytes. Proflavine, given its ability to intercalate DNA, prominently highlights nuclear structures within cells. Since human erythrocytes have little DNA content, there is very little proflavine signal present from these cells. While the erythrocytes are not visible with proflavine staining, the monolobar and multilobar structures within leukocytes are clearly distinguishable. Proflavine stained neutrophils exhibit classic multilobar architecture, with detectable granulation in the cytoplasm. Proflavine stained monocytes display a smooth, convoluted nuclear envelope and relatively homogeneous cytoplasm. Proflavine stained lymphocytes are easily distinguished by their characteristically smaller size, and display a relatively heterogeneous chromatin staining pattern and scant cytoplasmic labeling.

### Morphology and Quantitative Image Analysis of Oral Cells

Cytology images were analyzed quantitatively to extract important metrics indicative of cellular morphology. Both manual image metrics and automatic image texture analysis were used. Using ImageJ, the ratio between nuclear and cytoplasmic area was computed for comparison between normal human oral squamous cells and cultured Cal 27 oral squamous carcinoma cells. Using automated image texture analysis, we also report the use of entropy- a statistical measure of the randomness of pixel intensity within an image- to characterize images of proflavine-stained oral cells.

### Manual Segmentation for Nuclear to Cytoplasmic Ratio

**Fig 6:**
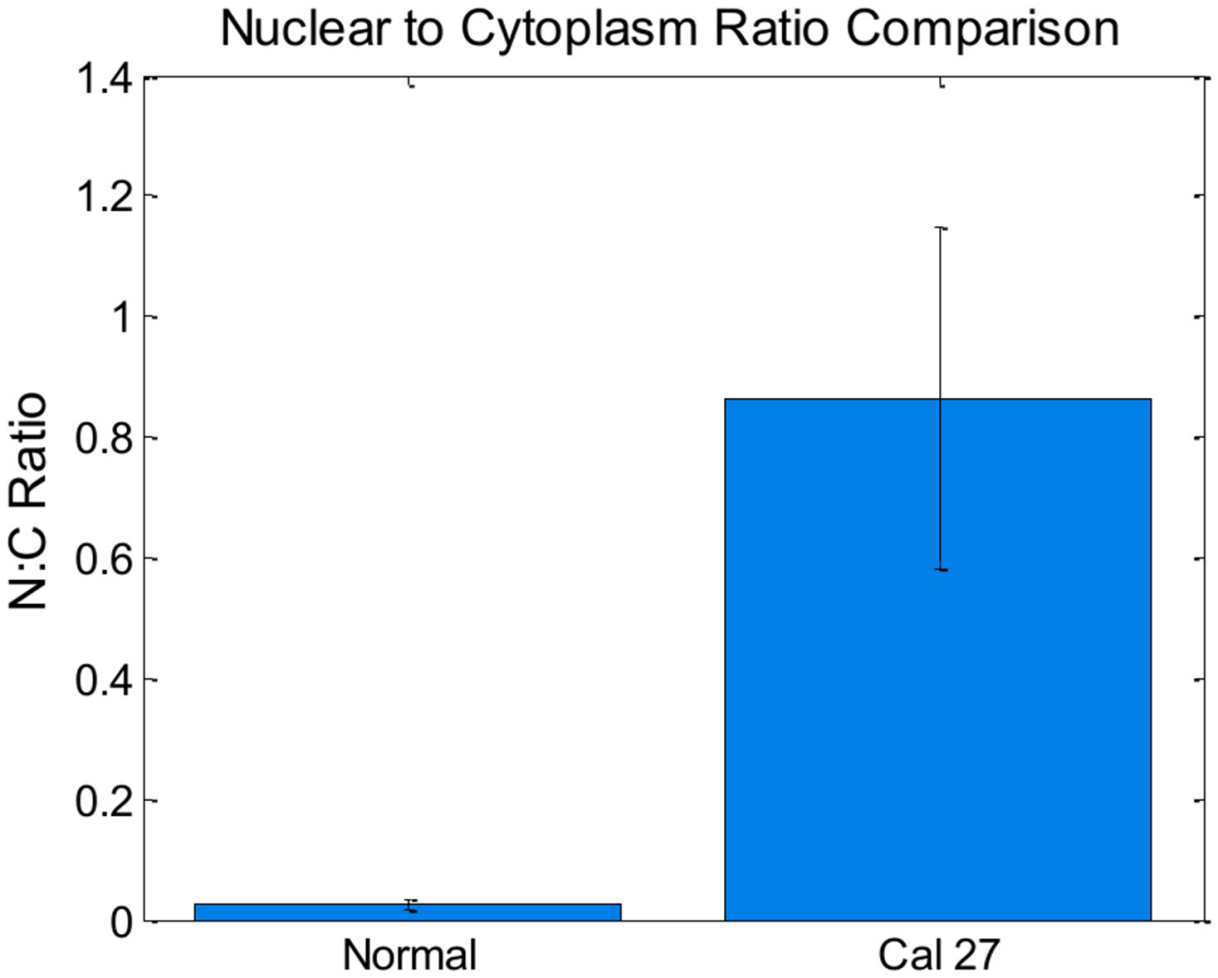
Bar plot comparing nuclear to cytoplasmic ratios for normal and CAL 27 cells. P value << 0.001 with Student T test. Error bars denote standard deviation.

The nuclei and cytoplasm of 100 normal cells and 100 CAL 27 cells were manually segmented and measured in ImageJ. Mean and standard deviation values for nuclear to cytoplasmic ratio were computed as described in the methods section. These results can be seen in Fig 6. Note that the nuclear to cytoplasmic ratio of normal oral cells was significantly smaller than the ratio of Cal 27 oral squamous carcinoma cells. This is expected when comparing normal, mature exfoliated oral squamous cells to *in vitro* cultured oral squamous carcinoma cells.

### Image Texture Analysis

**Fig 7:**
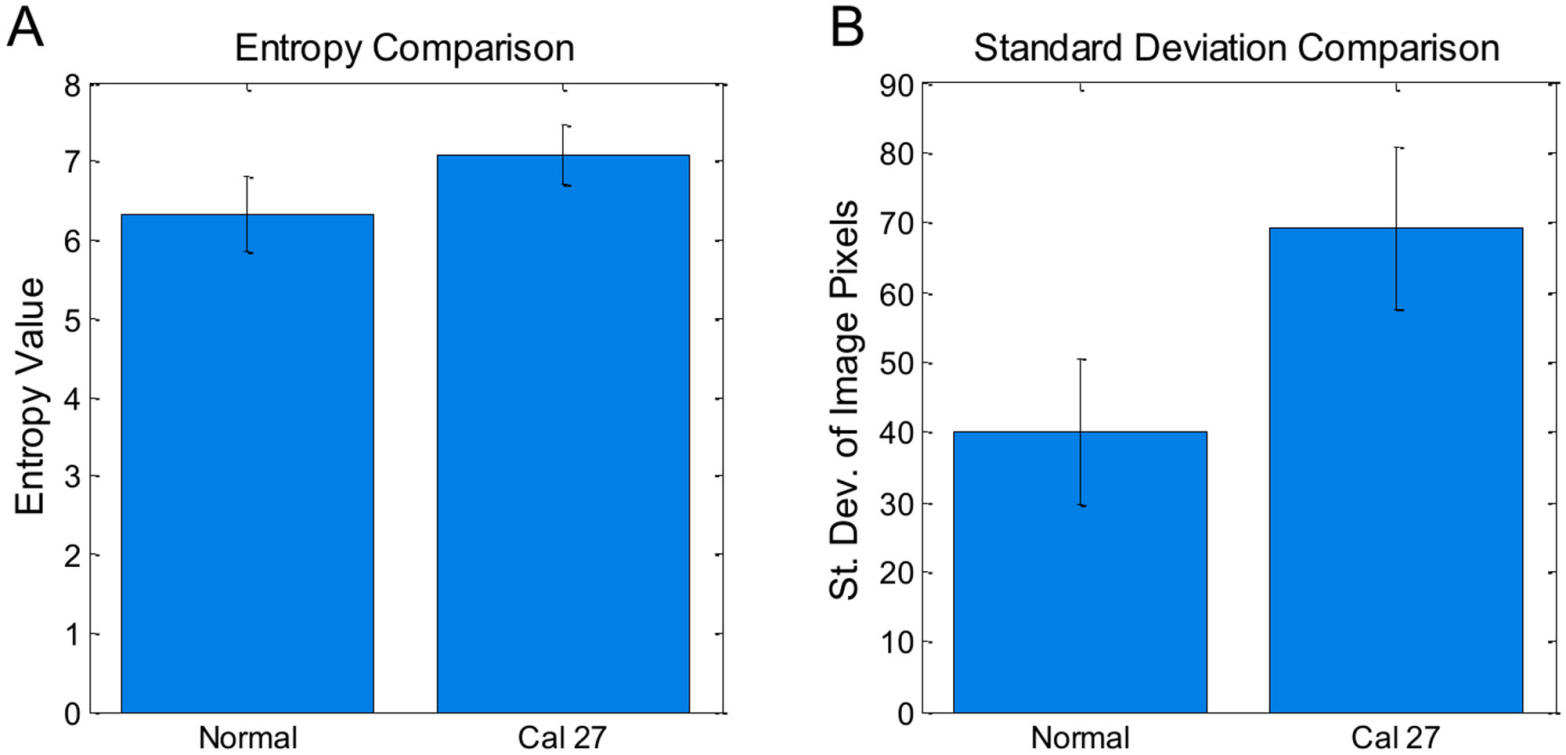
Bar plot comparing features between normal and CAL 27 cells. For both plots, p value << 0.001 with Student T test. 100 cells per group. Error bars denote standard deviation.

The same 200 images used to calculate nuclear to cytoplasmic ratios were analyzed using automated image texture analysis. The texture of each image was quantified individually using standard deviation and entropy. The difference in mean standard deviation and mean entropy values for each cell type were statistically significant (Fig 7). The average entropy (measure of pixel intensity randomness) values for the cultured CAL 27 carcinoma cells were significantly larger than the normal exfoliated oral cells. Similarly, the standard deviation of pixel values in the CAL 27 images were significantly larger than the normal exfoliated oral cells. These results imply that the internal texture, or variation in pixel intensity values within the image of each type of cell, is significantly greater in the Cal 27 cells when compared to the normal oral squamous cells.

## Discussion

In this manuscript, we have demonstrated that proflavine can be used to rapidly stain fresh cells for cytologic analysis. Proflavine is an effective fluorescent contrast agent for exfoliated oral squamous cells, highlighting nuclear and cytoplasmic structures, including keratohyalin granules in mature cells. Structural definition in proflavine-stained cells is comparable to Giemsa or Papanicolaou staining. As opposed to the multi-step procedure of Papanicolaou staining, proflavine staining requires only the addition of the dye to the cell medium before mounting on a slide, eschewing the need for fixation. Giemsa is also a conveniently applied cytologic stain for hematology specimens, but lacks the benefit of the high signal to background ratios that fluorescence imaging provides. Compared to traditional pathology stains, proflavine is cost-effective, requires little setup and materials, and does not require lengthy staining time (see Table 1). Additionally, proflavine can rapidly stain freshly collected leukocytes, clearly showing the multilobar structure of granulocytes in contrast to other cellular components. Finally, fluorescence images of proflavine stained cells can be analyzed quantitatively to highlight statistically significant differences in cell types based on morphologic features.

**Table 1:** Comparison of cost and time for pathology stains.

	Proflavine	Giemsa	Diff-Quik	H&E
**Cost/Slide** (materials only)	Estimated $0.50	Estimated $1.00†	$1.50 [25]	$3.39 [26]
**Time/Slide** Unit Value*	Estimated 1 min	14 min [27]	10–30min [25]	1.5 min‡ [27]

*Not including time required to examine a slide to reach a diagnosis.

^†^Based on an average yield of 1000 slides/L

^‡^H&E performed with an autostainer.

Normal oral epithelial cells can be classified as basal, parabasal, intermediate, and superficial. Using our system we have collected mostly intermediate and superficial cells. Normal superficial cells have a large cytoplasm, with small, well defined nuclei, and small granules [4]. Cancer cells, due to their high mitotic activity, tend to have enlarged nuclei with diminished cytoplasm, to the point that the cytoplasm might visibly be a narrow region around the nucleus, or the nuclei aren’t clearly visible [4]. Of the 100 normal oral epithelial cells stained with proflavine, all exhibited clearly defined nuclei, while of the 100 cancer cells imaged, slightly more than half displayed clearly defined nuclei. Proflavine stained cells displayed comparable cytologic features to PAP stained cells, such as well-defined membranes, nuclei, granules, and bacteria. Tables 2 and 3 qualitatively compare key image features of proflavine stained cells with conventionally (PAP or Giemsa) stained cells. Twenty randomly selected images from each set were visually inspected and evaluated for contrast between features—membranes, nuclei, and keratohyalin granules—and the surrounding structures, and each set was given an overall rating of distinct / strong contrast (++), visible but moderate contrast (+), or poor contrast / not visible (-), with one feature (the presence of bacteria) classified as N/A for cultured CAL 27 cells which were intentionally kept free of bacteria (see Table 2).

**Table 2:** Comparison of proflavine and PAP stained oral cells.

Feature	Normal Oral		CAL 27	
	Proflavine	PAP	Proflavine	PAP
Membrane	+	++	++	++
Nucleus	++	++	+	+
Nucleolus	-	-	+	+
Keratohyalin granules	++	++	-	-
Golgi zone	-	-	++	+
Bacteria	++	+	N/A	N/A

**Table 3:** Comparison of proflavine and Giemsa stained leukocytes.

Feature	Neutrophils		Monocytes		Lymphocytes	
	Proflavine	Giemsa	Proflavine	Giemsa	Proflavine	Giemsa
Lobar structure	++	++	++	++	++	++
Membrane	++	++	+	++	+	++
Lobar texture	++	++	++	++	++	++

Clearly defined cell membranes (++) were defined as showing a sharp contrast between the edge of the membrane and the dark background surrounding the cell, while visible membranes that had indistinct borders were defined as visible but moderate contrast (+). PAP stained cells were designated as having clearly defined membranes (++) if they showed a sharp contrast between the bluish-purple edge of the membrane and the white-beige background surrounding the cell, while membranes that had a color difference but did not have a clearly defined boundary were defined as (+). As seen in Table 2, PAP stained cells have clearly defined cell membranes, and proflavine stained CAL 27 cells also had pronounced definition. Proflavine stained normal cells were labelled with visible with moderate contrast (+) due to some of the assessed cell membranes having low membrane to background contrast, or having a small gap in the perimeter, where the membrane was left unstained and the cytoplasm seems to merge with the background and surroundings. The nuclei, nucleoli, and bacteria were similarly assessed, with their outer boundary compared against its surroundings (cytoplasm, nucleus, or cytoplasm, respectively) for strong contrast (fluorescent or colorimetric). CAL 27 cells have large nuclei that can be difficult to distinguish due to their size and lack of sharp boundaries in both PAP and proflavine stained images, whereas their normal counterparts demonstrate high contrast with both methods of staining. Nucleoli, when visible, appeared with high contrast (++). The Golgi zone lacked the sharp boundaries of other features, and was defined as clear (++) when the area was a different intensity or color from the rest of the cytoplasm, visible but unclear (+) when there was a difference in the area surrounding the nucleus but difficult to tell where the Golgi zone ended and the cytoplasm began, and unclear (-) if the Golgi zone was not apparent, with proflavine showing a greater contrast in CAL 27 cells, but not distinguishable in either stain in normal oral cells.

Whole blood from healthy volunteers was also imaged, and classified by an expert pathologist (SGS). Features highlighted by proflavine staining, specifically the shape and number of lobar structures in the nuclei of neutrophils, could be used to classify leukocyte types [28]. Features such as lobar structure and membrane definition were comparable to Giemsa stained whole blood, with the primary difference being that erythrocytes are nearly invisible in proflavine fluorescence images. Randomly selected images from each set were assessed for features such as lobar structure, membrane definition, and lobar texture (see Table 3). Lobar structure and membrane was defined as high contrast (++) when the outer boundary was in sharp contrast with its surroundings, and the shape of the lobes or membrane were easy to identify. Both Giemsa and proflavine stained leukocytes showed clearly defined lobes, and did not require use of the visible but moderate contrast (+) label. Proflavine stained leukocytes had a higher tendency to have diffuse membrane boundaries (-), though they were clearly different from their surroundings. Lobar texture was assessed by looking at contrasting regions within the lobes themselves; areas within the same lobes can be in contrast (fluorescent or colorimetric) with neighboring regions. High contrast lobar texture (++) was defined as distinct patchy contrasting regions within lobes, while the lobe texture was deemed to have moderate contrast (+) if there was little diffuse contrast within. Lobes without internal contrast (-) were commonly due to stain saturation and were rare in both Giemsa and proflavine stained leukocytes. In summary, proflavine stained cells displayed comparable cellular features to PAP and Giemsa stained cells, with less membrane definition overall due to the preferentially nuclei staining nature of proflavine, but no significant loss of features that would limit cytological classification.

Our study included only a select set of examples of quantitative image analysis of proflavine stained cells. We have shown that quantification of morphological features of normal oral, abnormal oral, and leukocytes is achievable using proflavine-staining cytology. Computational classification algorithms have been previously used in conjunction with imaging to provide additional tools for the pathologist [29]. Such algorithms could be implemented in cytological analysis of proflavine-stained leukocytes, using a combination of image texture and automatically-segmented structural features. Quantitative image analysis could be useful as an adjunctive tool when analyzing cytology specimens which are ambiguous. Additionally, computer-aided analysis could allow for diagnosis in the absence of an expertly trained pathologist. Computer-aided diagnostic platforms are currently in use, including image segmentation and analysis systems like the CytoSavant and the CellSearch system (specifically, CellTracks Analyzer) [30,31]. Additional image analysis platforms and systems have been either implemented, or heavily researched, such as image analysis of ultrasound data [32,33,34].

As a research tool, proflavine staining could enable quantitative morphologic comparisons between many different cell types, while simplifying current imaging lab procedures. Proflavine fluorescence is compatible with conventional fluorescein isothiocyanate (FITC)-equipped microscopy tools, and could potentially be introduced as a label in flow cytometry. Other methods of flow cytometry / image cytometry include classification of lymphoblastic leukemia using specific cell surface markers through immunofluorescent labeling [35]. Non-specific dyes such as propidum iodide and FITC have been used in combination to distinguish contours, such as the nuclei, and can approximate the accuracy and sensitivity of “advanced flow cytometers” [36].

Proflavine, as an aminoacridine derivative, is a promising contrast agent for point-of-care cytology due to its rapid uptake, highly simplified staining procedure, and robust and consistent staining of cellular structures. Other aminoacridine derivatives, such as acridine orange- with a peak excitation wavelength of 450 and emission of 650- have also been widely studied as a nucleic acid stain [37]. Acridine orange has also been studied for point-of-care diagnostics, such as malaria, due to its rapid and selective staining and high contrast images of intracellular *plamsmodium* parasites [38,39]. Cost-effective point-of-care cytology is currently of high interest to the biomedical research community, especially for applications in rural or developing regions. Cell phone imaging technology has been developed and is still being investigated. For example, an inexpensive white blood cell density counter prototype measured fluorescence in leukocytes stained with Syto16, a fluorescent nucleic acid stain that required a 30 minute incubation period [40]. We propose that proflavine can be used as a fluorescent structural dye across a wide range of unfixed cytology and solid tissue specimens. Using proflavine in combination with current point-of-care diagnostics, including portable microscopy platforms, microendoscopy devices and lab-on-chip technology, could prove beneficial in reducing time, procedure, and cost of screening diseases such as cervical cancer [23,41,42,43].
